# Animal Allergens, Endotoxin, and β-(1,3)-Glucan in Small Animal Practices: Exposure Levels at Work and in Homes of Veterinary Staff

**DOI:** 10.1093/annweh/wxab053

**Published:** 2021-08-07

**Authors:** Eva Zahradnik, Ingrid Sander, Olaf Kleinmüller, Anne Lotz, Verena Liebers, Bente Janssen-Weets, Stéphanie Kler, Christiane Hilger, Alexandra Beine, Frank Hoffmeyer, Albert Nienhaus, Monika Raulf

**Affiliations:** 1 Department of Allergology and Immunology, Institute for Prevention and Occupational Medicine of the German Social Accident Insurance, Institute of the Ruhr-Universität Bochum (IPA), Bochum, Germany; 2 CVcare, Universitätsklinikum Hamburg-Eppendorf, Hamburg, Germany; 3 Department of Epidemiology, Institute for Prevention and Occupational Medicine of the German Social Accident Insurance, Institute of the Ruhr-Universität Bochum (IPA), Bochum, Germany; 4 Department of Infection and Immunity, Luxemburg Institute of Health, Esch-sur-Alzette, Luxemburg; 5 Department of Dermatology and Allergy Center, Odense Research Center for Anaphylaxis, University of Southern Denmark, Odense, Denmark; 6 Department of Medicine, Institute for Prevention and Occupational Medicine of the German Social Accident Insurance, Institute of the Ruhr-Universität Bochum (IPA), Bochum, Germany; 7 Department of Occupational Medicine, Hazardous Substances and Health Research (AGG), Institution for Statutory Accident Insurance and Prevention in the Health and Welfare Services (BGW), Hamburg, Germany

**Keywords:** animal allergens, cat, dog, endotoxin, β-(1,3)-glucan, guinea pig, horse, occupational exposure, rabbit, veterinary practice

## Abstract

**Objectives:**

In veterinary settings, high exposures to animal allergens and microbial agents can be expected. However, occupational exposure levels are largely unknown. The objective of this study was to estimate the allergen, endotoxin, and β-(1,3)-glucan concentrations in small animal practices and in the homes of practice employees.

**Methods:**

Dust samples were collected using electrostatic dust fall collectors in diverse rooms of 36 small animal practices, as well as in employees’ homes. Major animal allergens (Fel d 1, Can f 1, Ory c 3, Cav p 1, Equ c 1, Bos d 2), domestic mite (DM) allergens, and β-(1,3)-glucan levels were measured using enzyme immunoassays. Endotoxin was determined using the *Limulus* amoebocyte lysate assay. Influences on exposure levels were analyzed using multilevel models.

**Results:**

The levels of Can f 1, Fel d 1, Ory c 3, and Cav p 1 were up to 30 times higher in practices compared with homes without animals, but significantly lower compared with the homes with the respective pet. Although horses were not treated in the practices, Equ c 1 was found in 87.5% of samples, with the highest concentrations measured in changing rooms. DM levels were significantly lower in practices than in all private homes, and endotoxin levels were similar to those in homes with pets. In the practice itself, exposure levels were significantly influenced by animal presence, type of the room, and area per employee; whereas, room volume and diverse cleaning measures had mostly no effect.

**Conclusions:**

Exposure to animal allergens is high in veterinary practices, but it does not reach levels of households with pets. Domestic mite allergen and endotoxin exposure seem to be low for workers in veterinary practices. The high Equ c 1 detection rate strongly indicates dispersal of allergens, most likely through clothing and hair.

**What’s Important About This Paper?**

This is the first study to compare the levels of exposure to diverse animal allergens, endotoxin, and β-(1,3)-glucan between the workplace and homes of German veterinary practice employees. Our study shows that although animal allergen levels were high in practices, they were lower than those measured in homes of pet owners. Interestingly, allergen levels were also high for animals not treated in the practices, e.g. horses, most likely due to passive transfer via clothing. Domestic mite allergen and endotoxin exposure seems to be low for workers in veterinary practices. In addition, this is the first study presenting the results of major allergens from rabbits (Ory c 3) and guinea pigs (Cav p 1) measured using newly developed immunoassays.

## Introduction

Exposure to animal allergens is a relevant risk factor in the development of sensitization, and allergic diseases (Konradsen *et al.*, 2015). Animal allergies tend to affect the general population mainly due to the large number of pet owners. Contact with animals also represents an occupational health hazard. Both the prevalence of allergic diseases and allergen (Jones, 2015) levels have been well investigated for laboratory animal workers handling mice and rats, and cattle farmers (Heutelbeck *et al.*, 2007; Zahradnik *et al.*, 2011; Schlünssen *et al.*, 2015). Interestingly, although veterinarians are among those most exposed to animal allergens, only a few studies have investigated allergic symptoms among veterinary staff. For example, 40% of California veterinarians reported animal-related respiratory and/or skin symptoms. The most commonly reported causes of symptoms were cats (26%), dogs (19%), horses (7%), and cattle (7%) (Susitaival *et al.*, 2003). In a Canadian survey, 39% of the study participants developed allergies during their veterinary career, and the most commonly reported allergy triggers were hair and dander from companion animals (Epp and Waldner, 2012). In Germany, the ‘Berufsgenossenschaft für Gesundheitsdienst und Wohlfahrtspflege’ (BGW), a part of the German Social Accident Insurance, provides a mandatory service for all veterinary practices in order to identify the most important health risks to veterinarians and their staff. According to their database, allergic reactions to animals (respiratory symptoms or allergic contact dermatitis) accounted for 23.8% of all verified occupational diseases in this group (Nienhaus *et al.*, 2005).

The exposure levels to animal allergens in veterinary practices are largely unknown. Thus far, only one study has been published examining allergen exposure in a companion animal hospital (Samadi *et al.*, 2010). In contrast, there are numerous publications on the quantitative measurements of diverse animal allergens [dog (Can f 1), cat (Fel d 1), mouse (Mus m 1), horse (Equ c 1)] in homes, schools, and other public places (Zahradnik and Raulf, 2014). In general, exposure to animal allergens occurs in every type of indoor environment, even in locations where no animals reside. In addition, there is strong evidence that human clothing and hair are the primary means by which allergens are transferred (Lucca *et al.*, 2000; Krop *et al.*, 2006).

Allergen concentrations vary considerably among different environments and are dependent on numerous factors. For example, in addition to animal presence, differences in allergen concentrations are associated with the number of pet owners and building-related factors, such as size and type of room, type of flooring and furnishing, cleaning frequency, and ventilation system (Zahradnik and Raulf, 2014).

No data are currently available on allergen exposure to guinea pigs, due to a lack of quantification assays. Several guinea-pig allergens have been characterized, with Cav p 1 being the major allergen (Hilger *et al.*, 2011; Swiontek *et al.*, 2021). Exposure to rabbit allergens has been performed only once in settled dust from homes and airborne samples from animal facility using an immunoassay for Ory c 1 (Willerton and Mason, 2018). Another major rabbit allergen Ory c 3 structurally related to Fel d 1 has been isolated and characterized by (Hilger *et al.*, 2014). Recently, new assays have been developed to measure Cav p 1 and Ory c 3, which were used for the first time in this study.

Exposure to microbial components, such as endotoxins (part of the outer membrane of Gram-negative bacteria) and β-(1,3)-glucans (part of fungal cell walls) are considered potential health hazards in the field of veterinary medicine. Exposure of animal farmers to elevated levels of these inflammatory agents was associated with allergic and non-allergic respiratory effects, and is proposed to induce similar health effects among veterinarians (Samadi *et al.*, 2013). Endotoxin levels were found to be low in veterinary practices with companion animals (Samadi *et al.*, 2010). β-(1,3)-glucan levels have not yet been investigated.

Therefore, the aim of this study was to characterize exposure levels to major allergens from mammals (Can f 1, Fel d 1, Ory c 3, Cav p 1, Equ c 1, Bos d 2), domestic mite (DM) allergens, endotoxin, and β-(1,3)-glucan in small animal veterinary practices. In addition, these biological agents were also measured in the homes of practice employees to compare home and work environment.

## Methods

### Study design

The study was conducted in 36 small animal practices across North Rhine–Westphalia, Germany, and in 101 of their employees’ homes from October 2017 until February 2019. Dust sampling and sampling documentation (i.e. duration, position and height of sampling equipment, room size, room ventilation) were carried out in the practices by a professional field worker, who tried to include all available rooms during sampling. The rooms (*n* = 304) were grouped into 11 categories: reception/waiting room, examination room, X-ray/ultrasound room, operating room, surgery preparation room, inpatient ward, pharmacy/laboratory, storage/utility room, break room, office, changing room. Short questionnaires were used to collect information about practice characteristics (location, size, number of rooms, number of employees, opening hours), type and percentage of treated animals, and type and frequency of cleaning measures. Dust samples were collected at home by the study participants, who received detailed instructions on how to use electrostatic dust fall collectors (EDCs) in rooms they occupied the most. They were then required to fill a questionnaire addressing sampling characteristics, as well as information on the presence of pets (cat, dog, rabbit, guinea pig, hamster) at home, and any direct contact to animals during leisure activities.

This study was part of the project AllergoMed which was approved by the Ethics Committee of the Ruhr University Bochum in Germany (registration number: 17-6022). Participation in the project was voluntarily and all participants signed an informed consent before taking part in the study.

### Dust sampling

Dust sampling was performed using EDCs consisting of a polypropylene folder with two dust-binding cloths (Techmed-Textil-Service-GmbH, Dipperz, Germany), each with a surface exposure area of 0.0209 m^2^. The cloths were made pyrogen-free by heating for 4 h at 200°C. In most cases, the EDCs were left in a horizontal position at a median height of 1.9 m (range: 1–2.6 m) above the floor for the recommended time of 14 days (range: 7–36 days) to collect settling airborne dust. After the sampling period, the EDCs were closed, individually placed into Ziploc bags and sent to the laboratory by regular mail in a pre-addressed envelope. Once received, one cloth from each EDC was removed from the folder under a sterile workbench, transferred to autoclaved 150 ml beakers and stored at 4°C until extraction of endotoxin. The second cloth was frozen in the polypropylene folder, placed in a Ziploc bag overnight at −20°C to eliminate mite proliferation on the cloth, and then stored at room temperature until extraction of allergens and β-(1,3)-glucan.

### Extraction

Allergens and β-glucans were sequentially extracted from the cloths in 15 ml phosphate-buffered saline (PBS), pH 7.4 with 0.05% Tween 20 (PBST) by rotation for 1 h at room temperature. After removing the cloths, the extracts were centrifuged at 3000 g for 15 min. The supernatants were stored in aliquots at −80°C until allergen analysis. For the β-(1,3)-glucan analysis, the pellets were re-suspended with 2 ml of the supernatant and autoclaved at 121°C and 1 bar for 20 min. These autoclaved resuspensions were centrifuged at 3000 g for 15 min and stored in aliquots at −80°C.

Endotoxin was extracted from the second EDC cloth in 20 ml pyrogen-free water (*Aqua ad iniectabilia*, DeltaSelect, Reutlingen, Germany) by shaking (160 shakes min^−1^) for 1 h at room temperature. The extracts were transferred into pyrogen-free tubes and centrifuged at 1000 g for 10 min. The supernatants were stored in aliquots at −80°C until analysis.

### Quantification of allergens, endotoxin, and β-(1,3)-glucan

Allergen levels of Fel d 1, Can f 1, and Bos d 2 were determined using monoclonal antibodies and calibration standards purchased from Indoor Biotechnologies Inc. (Charlottesville, VA, USA) according to protocols described previously for cat and dog (Sander and Lotz *et al.*, 2016) and for cattle (Zahradnik *et al.*, 2015). Equ c 1 concentrations were quantified using an immunoassay based on polyclonal antibodies and naturally-purified Equ c 1 as a standard (Zahradnik *et al.*, 2018). To improve assay sensitivity, the chromogenic substrate ABST [2,2′-azino-bis(3-ethylbenzothiazoline-6-sulfonic acid)] was exchanged with the fluorogenic substrate QuantaBlu (ThermoScientific, Rockford, IL, USA). A sensitive immunoassay based on polyclonal antibodies to *Dermatophagoides farinae* extract was used to estimate domestic mite levels. Due to strong cross-reactivity, this assay detects allergens from several house dust and storage mite species (Sander *et al.*, 2012). β-(1,3)-Glucan measurements were performed as previously described by (Sander *et al.*, 2008). Endotoxin was determined using a kinetic chromogenic limulus amoebocyte lysate assay (Charles River, Sulzfeld, Germany) according to the manufacturer’s instructions.

For the detection of Ory c 3 and Cav p 1, newly developed enzyme immunoassays were applied. The production of recombinant proteins and polyclonal antibodies (pAb) is described in the supplementary material provided online. Briefly, 384 well microtiter plates (Nunc MaxiSorp, ThermoFischer Scientific, Waltham, Massachusetts) were coated with purified anti-Ory c 3 or anti-Cav p 1 pAb at 0.5 µg ml^−1^ in PBS (50 µl well^−1^) overnight at 4°C, followed by a blocking step with 3% bovine serum albumin in PBST (100 µl well^−1^). Dust samples were added in duplicate to the microplates (undiluted and diluted ½ in PBS). Standard curves were established using a native Ory c 3 purified from rabbit hair with concentrations ranging from 0.01 to 50 ng ml^−1^, or a mixture of rCav p 1 isoallergens (0.001 to 50 ng ml^−1^) at 50 µl well^−1^. Bound allergens were quantified using biotinylated anti-Ory c 3 or anti-Cav p 1 pAb diluted 1/2000 in blocking buffer (50 µl well^−1^) followed by incubation with horse-radish peroxidase labeled streptavidin (Fitzgerald, Concord, MA, USA) diluted 1/20 000 in blocking buffer. All incubations were carried out for at least 1 h at room temperature whilst shaking, followed by three washes with PBST between successive steps. The assays were developed using QuantaBlu.

Values below the lower limit of detection (LOD) were replaced by 2/3 LOD. All values above the LOD were divided by the number of sampling days and multiplied by 14 to adjust the values to the recommended dust sampling duration of two weeks. This was done because allergen levels increase proportionally with deployment time over a period of 4 weeks (Sander and Lotz et al., 2016). All values were then calculated as ng m^−2^ or EU m^−2^. The LODs for EDC samples were 5.7 ng m^−2^ for Cav p 1, 7.2 ng m^−2^ for Fel d 1, Can f 1, and Equ c 1, 14.4 ng m^−2^ for Bos d 2, 16.5 ng m^−2^ for Ory c 3, 35.9 ng m^−2^ for DM, and 95.7 ng m^−2^ for β-(1,3)-glucan.

### Statistical analysis

The concentrations of all analytes were log-transformed and analyzed using multilevel-level models with sample as level-one unit and practice as level-two unit to determine (i) differences between home and practice environments, (ii) differences between rooms with and without animals, and (iii) influencing factors on exposure levels within the practices. For the latter, independent variables in the models were: room type, room ventilation (by window), frequency of cleaning (wiping, sweeping, vacuuming), opening hours, room volume, and area per employee. Additionally, for each model the conditional intraclass correlation coefficient (ICC) was calculated, which is a measure of the degree of within-group homogeneity or between-group heterogeneity after controlling for contextual variables (Wang *et al.*, 2011). The ICC approaches one when the between-practice variation is very large relative to the within-room variation, indicating that samples collected from one practice are similar. Conversely, ICC approaches zero when the grouping of samples by practice conveys no additional information. Each analyte was analyzed in a separate model.

Statistical analyses were conducted using SAS, version 9.4 (SAS Institute, Inc. Cary, NC). If some of the exposure levels were below the limit of detection, the multilevel-models with a censored dependent variable were estimated as previously described (Vaida and Liu, 2009), and calculated using software R (R Core Team, 2015). The descriptive statistics and graphs were made with GraphPad Prism version 8.4.3 (GraphPad Software, Inc., La Jolla, CA).

## Results

### Exposure levels in practices and homes

Characteristics of the veterinary practices and homes of their employees are given in Table 1. In practices, Can f 1 was detected in all, Fel d 1 in 99.7%, Ory c 3 in 81.6%, Cav p 1 in 82.9%, and DM allergens in 64.8% of the samples. Although horses and cattle were not treated in practices, Equ c 1 was found in 87.5% and Bos d 2 in 22% of the samples. In homes, 92.1% of samples contained Can f 1, 79.2% Fel d 1, 18.8% Ory c 3, 50.5% Cav p 1, and 88.1% DM allergens. Moreover, Equ c 1 was found in 82.2% and Bos d 2 in 20.8% of the samples. All samples were above the limit of detection for endotoxin and β-(1,3)-glucan.

**Table 1.** Characteristics of practices and homes of practice employees.

Characteristic	Practices (*n* = 36)		
	Median	IQR	Range
Size of the practice	145 m^2^	112.5–204.5 m^2^	80–420 m^2^
Number of rooms	9	7–12.5	3–18
Size of the rooms	15 m^2^	12–20 m^2^	4–50 m^2^
Height of the rooms	2.5 m	2.5–2.9 m	2–4 m
Number of employees	5	4–8	1–14
Opening hours	29 h week^−1^	24–37 h week^−1^	18–53 h week^−1^
Treated animal species			
Dogs	40%	40–45%	35–60%
Cats	40%	35–45%	24–50%
Rabbits	8%	5–10%	0–20%
Guinea pigs	5%	3–9.75%	0–10%
Hamster	1%	0–2%	0–5%
Other animals*	0%	0–1%	0–5%
Cleaning measures	(*n*, %)		
Wiping every day	24 (66)		
Wiping less than every day	12 (33)		
Vacuuming every day	27 (75)		
Vacuuming less than every day	9 (25)		
Sweeping every day	16 (44)		
Sweeping less than every day	20 (56)		
	Homes (*n* = 101)		
	Median	IQR	Range
Number of rooms	4	3 - 6	2 - 20
Number of residents	2	2 - 3	1 - 5
Sampling rooms			
Size	22 m^2^	18–30 m^2^	9–60 m^2^
Floor level	1	0–2	-1–6
Carpet covering	0%	0–17.5%	0–100%
Type of the room	**(%)**		
Living room§	81		
Bedroom	17		
Kitchen	1		
Office	1		
Pets in the home	(%)		
None	20		
Dogs	51		
Cats	38		
Rabbits	4		
Guinea pigs	4		
Hamster	1		
Other animals$	8		
Contact to animals (outside the home during leisure time)	**(%)**		
None	10		
Dogs	76		
Cats	51		
Horses	24		
Cattle	3		
	Homes (*n* = 101)		
	Median	IQR	Range
Rabbits	14		
Guinea pigs	7		
Hamster	4		
Other animals^#^	4		

*other rodents, birds, reptiles.

^$^gerbil, squirrel, mice, frogs, fish, geckos, axolotl, corn snake, aquatic turtles.

^#^ birds, mice, goats, sheep.

^§^ including mixed use (living room/bedroom, kitchen-living room, living room/office).

In order to classify the levels of allergen and endotoxin exposure in the practices, concentrations were compared with those in employees’ homes (Table 2), which were grouped according to the presence of the specific animal, or the presence of pets for DM and endotoxin. In the case of Equ c 1 and Bos d 2, homes were classified according to whether employees had contact with horses or cattle during their leisure time. In general, analytes’ concentrations varied widely (up to three orders of magnitude). Allergens levels of the animals treated at practices (Can f 1, Feld 1, Ory c 3, Cav p 1) were significantly higher (up to 30 times) in the practices compared with homes where the animals were not present, but significantly lower (except Ory c 3) compared with the homes with the respective animals. Although not significant, Ory c 3 levels were higher in homes with rabbits compared with levels measured at practices. A similar trend was observed for Equ c 1 where compared with the practices, 11-fold higher Equ c 1 levels were found at the homes of employees who were in contact with horses outside of work. Conversely, employees with no contact to horses outside of work exhibited 6-fold lower median Equ c 1 levels compared with those in the practices. DM levels in the practices were significantly lower than in households with and without pets. Furthermore, endotoxin levels did not differ between practices and homes with pets, and were twice as high in practices compared with homes without pets, albeit not significant.

**Table 2.** Allergen, endotoxin, and β-(1,3)-glucan levels in small animal practices and homes of practice employees.

	*N*	ND	Median	IQR	Range	*P*-value^#^
Can f 1 (ng m^−2^)						
Practices	304	–	793	300–1606	9-10967	
Homes with dogs	52	–	1416	563–2163	16-17084	**0.0193**
Homes without dogs	49	8	34	11–73	<LOD-874	**<0.0001**
Fel d 1 (ng m^−2^)						
Practices	304	1	440	180–1058	<LOD-41254	
Homes with cats	38	–	1617	362–9009	59-166238	**<0.0001**
Homes without cats	63	21	15	<LOD-52	<LOD-324	**<0.0001**
Ory c 3 (ng m^−2^)						
Practices	304	56	282	50–81	<LOD-13880	
Homes with rabbits	4	–	973	230–4342	32–5417	0.2558
Homes without rabbits	97	82	<LOD	<LOD - <LOD	<LOD-1070	**<0.0001**
Cav p 1 (ng m^−2^)						
Practices	304	52	93	23- 257	<LOD-4427	
Homes with guinea pigs	4	–	3972	900–6236	134–6732	**0.0011**
Homes without guinea pigs	97	50	<LOD	<LOD- 32	<LOD-496	**<0.0001**
Equ c 1 (ng m^−2^)						
Practices	304	38	77	17–325	<LOD-8781	
Homes of employees with contact to horses	24	–	877	136–2058	20-62009	**<0.0001**
Homes of employees without contact to horses	77	18	12	7–28	<LOD-1258	**<0.0001**
Bos d 2 (ng m^−2^)						
Practices	304	237	<LOD	<LOD - <LOD	<LOD-128	
Homes of employees with contact to cattle	3	1	21		<LOD-456	n.a.
Homes of employees without contact to cattle	98	79	<LOD	<LOD - <LOD	<LOD-131	n.a.
DM (ng m^−2^)						
Practices	304	107	60	<LOD-163	<LOD-2597	
Homes with pets	80	8	157	61–408	<LOD-84292	**<0.0001**
Homes without pets	21	4	127	40–261	<LOD-7614	**0.0495**
Endotoxin (EU m^−2^)						
Practices	304	–	140	72–281	23–3362	
Homes with pets	79	–	164	67–434	20–6512	0.0897
Homes without pets	21	–	64	39–185	16–1380	0.0839
β-(1,3)-glucan (ng m^−2^)*						
Practices	300	–	2313	1341–3720	200–23572	

*N*: number of measurements; ND: number of not detectable samples; IQR: interquartile range; LOD: limit of detection; n.a.: not applicable.

#*P*-value in comparison to practices (determined in two-level-model), *P*-values <0.05 are printed in bold

*β-(1,3)-Glucan was determined only in EDC-samples from practices.

### Exposure levels in practices classified by room type

**Figure 1.**
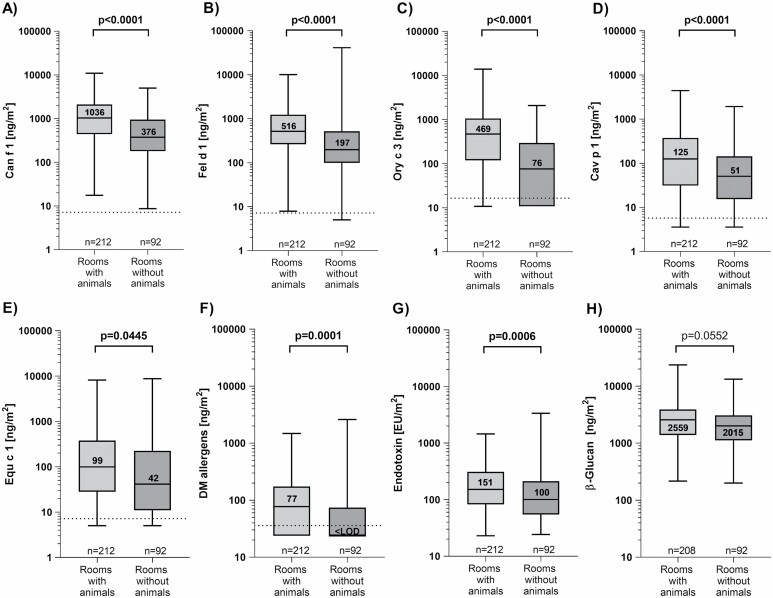
Levels of Can f 1 (A), Fel d 1 (B), Ory c 3 (C), Cav p 1 (D), Equ c 1 (E), DM allergen (F), endotoxin (G), and β-(1,3)-glucan (H) in veterinary practice rooms categorized by animal presence. Numbers within the boxes represent the median values. The LODs are marked with a dotted line.

Concentrations of all allergens, endotoxin, and β-(1,3)-glucan were sorted according to room type (Table 3), and classified as rooms with or without animals (Fig. 1). A detailed overview of the Bos d 2-results is not provided due to the large percentage of samples below the LOD and very low concentrations compared with the other allergens (maximum 128 ng m^−2^). The levels of all analytes, except β-(1,3)-glucan were significantly higher in rooms with animals compared with those without, but these differences were small. For example, Can f 1, Fel d 1, and Cav p 1 concentrations only differed by about 2.5-fold. Of all allergens, the highest levels were found for Can f 1 followed by Fel d 1, and the lowest for DM allergens. Can f 1 values were on average twice as high as Fel d 1 and 6-fold higher than Cav p 1. Differences were also found among the individual room types following a similar pattern for all determinants, except Ory c 3 and Equ c 1. In rooms occupied by animals, the highest allergen concentrations were found in the examination room and the lowest in the operating room. In contrast, the highest concentrations of Ory c 3 and Equ c 1 were measured in the inpatient ward and reception/waiting area, respectively. Changing rooms had the highest allergen, endotoxin and β-(1,3)-glucan levels among those where no animals were present. Interestingly, Equ c 1 levels were much higher in changing rooms than in rooms with animals (Table 3).

**Table 3.** Allergen, endotoxin, and β-(1,3)-glucan levels in different room types of small animal practices.

Room category		Can f 1 (ng m^−2^)		Fel d 1 (ng m^−2^)		Ory c 3 (ng m^−2^)		Cav p 1 (ng m^−2^)		Equ c 1 (ng m^−2^)		Domestic mite (ng m^−2^)		Endotoxin (EU m^−2^)		β-Glucan (ng m^−2^)		
	*N*	*M*	Range	*M*	Range	*M*	Range	*M*	Range	*M*	Range	*M*	Range	*M*	Range	*N*	*M*	Range
**Rooms with animals**																		
Examination room	61	2184	21-10967	1377	14–6374	723	<LOD-5099	400	<LOD-4427	137	<LOD-4128	161	<LOD-1126	247	28–1069	59	3181	506–23572
Reception/waiting room	58	1193	142–10743	438	9-7939	286	<LOD-4770	107	<LOD-1463	180	<LOD-8137	93	<LOD-1481	200	36–1453	56	3248	329–22564
Operating room	33	399	18–6918	210	8-7587	324	<LOD-2548	41	<LOD-1874	42	<LOD-565	28	<LOD-665	84	28–952	33	1628	216–3721
X-ray-/ultrasound room	31	733	60–2973	405	19–1478	206	<LOD-1306	57	<LOD-860	133	<LOD-1112	52	<LOD-217	99	23–856	31	2046	714–5801
Inpatient ward	16	476	102–2288	859	32–2719	1198	<LOD-13880	173	<LOD-1041	35	<LOD-629	45	<LOD-599	117	42–726	16	2102	590–5476
Surgery preparation	13	830	166–1303	671	163–10031	913	<LOD-2465	137	<LOD-500	79	<LOD-1020	58	<LOD-359	120	46–309	13	2158	395–6085
**Rooms without animals**																		
Pharmacy/laboratory	30	307	38–2290	173	9-1252	71	<LOD-2078	39	<LOD-1927	41	<LOD-1181	<LOD	<LOD-2597	73	24–600	30	2165	432–7420
Break room	28	855	60–5000	373	32–2835	120	<LOD-1843	97	<LOD-637	129	<LOD-8781	47	<LOD-428	161	36–3362	28	2437	575–8274
Storage/utility room	17	212	9-3276	125	<LOD-624	52	<LOD-969	19	<LOD-123	14	<LOD-1069	<LOD	<LOD-558	53	29–566	17	1468	200–7141
Office	9	184	15–1909	160	50–661	<LOD	<LOD-680	32	<LOD-211	11	<LOD-254	<LOD	<LOD-745	81	55–574	9	1863	841–2223
Changing room	8	901	46–1531	566	41-41254	356	<LOD-728	176	<LOD-492	589	<LOD-6764	83	<LOD-376	259	67–699	8	3551	769–13260

*N*: number of measurements, *M*: median, LOD: limit of detection.

### Influences on exposure levels in practices

Analyte concentrations in the practices were significantly influenced by the room type in the multilevel models (Table 4), confirming the results of the descriptive analysis. In general, the examination room had significantly higher concentrations than the majority of other room types. Equ c 1 was the only allergen where the concentration in the examination room was significantly lower compared with the changing room. The second factor that strongly and significantly influenced exposure levels was the area per employee. The more space available per employee, the lower the allergen and β-(1,3)-glucan exposure. However, this effect was not significant for endotoxin and Cav p 1. Less frequent ventilation led to reduced analyte concentration, which was only significant for Fel d 1, endotoxin, and β-(1,3)-glucan. Cleaning measures, opening hours and room volume had no influence on concentrations of analytes with the exception of DM allergens. In particular, increasing room volume was associated with reduced allergen levels of DM. The highest conditional ICC value was achieved for Equ c 1 (0.76) indicating that about 76% of the total variance in the outcome measure was due to variations between practices. However, no specific practice had an influence on DM allergen concentrations (ICC = 0.001).

**Table 4.** Influences on allergen, endotoxin, and β-(1,3)-glucan levels in small animal practices determined by multilevel models.

		*Can f 1*		*Fel d 1*		*Ory c 3*		*Cav p 1*		*Equ c 1*		*Domestic mite*		*Endotoxin*		*β-(1,3)-Glucan*		
	*N*	exp(*β*)	*P-*value	exp(*β*)	*P-*value	exp(*β*)	*P-*value	exp(*β*)	*P-value*	exp(*β*)	*P-value*	exp(*β*)	*P-value*	exp(*β*)	*P-value*	*N*	exp(*β*)	*P-value*
Intercept (ng m^−2^)		3809.815	**<.0001**	4722.467	**<.0001**	5941.929	**<.0001**	432.395	**<.0001**	152.359	**<.0001**	123.889	**<.0001**	266.218	**<.0001**		4365.708	**<.0001**
Room type																		
Examination Room	61	1.000		1.000		1.000		1.000		1.000		1.000		1.000		59	1.000	
Reception/waiting room	58	0.632	**0.0066**	0.388	**<.0001**	0.354	**<.0001**	0.352	**<.0001**	1.259	0.2808	0.590	0.0206	0.999	0.9923	56	0.934	0.5859
Operating room	33	0.189	**<.0001**	0.310	**<.0001**	0.412	**0.0015**	0.180	**<.0001**	0.282	**<.0001**	0.393	**0.0005**	0.482	**<.0001**	33	0.439	**<.0001**
X-ray-/ultrasound room	31	0.341	**<.0001**	0.420	**0.0001**	0.393	**0.0022**	0.296	**0.0001**	0.450	**0.0079**	0.554	**0.0351**	0.521	**0.0004**	31	0.762	**0.0778**
Inpatient ward	16	0.232	**<.0001**	0.771	0.3379	0.900	0.7742	0.478	**0.0416**	0.226	**<.0001**	0.564	0.1008	0.565	**0.0098**	16	0.607	**0.0077**
Surgery preparation	13	0.360	**0.0002**	0.790	0.4178	0.936	0.8645	0.367	**0.0091**	0.369	**0.0041**	0.510	0.0569	0.538	**0.0088**	13	0.584	**0.0073**
Pharmacy/laboratory	30	0.173	**<.0001**	0.202	**<.0001**	0.156	**<.0001**	0.191	**<.0001**	0.332	**<.0001**	0.280	**<.0001**	0.403	**<.0001**	30	0.709	**0.0261**
Break room	28	0.445	**<.0001**	0.377	**<.0001**	0.154	**<.0001**	0.218	**<.0001**	0.827	0.4672	0.506	**0.0148**	0.789	0.1846	28	0.716	**0.0285**
Storage/utility room	17	0.109	**<.0001**	0.115	**<.0001**	0.087	**<.0001**	0.120	**<.0001**	0.233	**<.0001**	0.169	**<.0001**	0.396	**<.0001**	17	0.482	**0.0001**
Office	9	0.136	**<.0001**	0.275	**0.0002**	0.064	**<.0001**	0.138	**<.0001**	0.200	**0.0001**	0.362	**0.0353**	0.518	**0.0190**	9	0.622	**0.0457**
Changing room	8	0.399	**0.0090**	1.179	0.6626	0.281	**0.0148**	0.365	**0.0451**	2.473	**0.0468**	1.597	0.3023	0.997	0.9915	8	1.200	0.4784
Ventilation																		
daily	169	1.000		1.000		1.000		1.000		1.000		1.000		1.000		168	1.000	
occasionally	76	0.809	0.1346	0.722	**0.0313**	0.990	0.655	0.794	0.2738	0.887	0.5206	0.906	0.5891	0.808	0.0807	73	1.009	0.9339
never	59	0.790	0.1675	0.682	**0.0348**	0.715	0.199	0.731	0.2196	0.820	0.3844	0.671	0.0684	0.737	**0.0381**	59	0.772	**0.0335**
Wiping																		
every day	202	1.000		1.000		1.000		1.000		1.000		1.000		1.000		198	1.000	
less than every day	102	1.444	0.2021	1.250	0.3885	0.460	0.1122	0.546	0.2649	2.055	0.2148	1.028	0.8903	1.165	0.4652	102	1.073	0.6483
Vacuuming																		
every day	218	1.000		1.000		1.000		1.000		1.000		1.000		1.000		214	1.000	
less than every day	86	0.795	0.4473	0.874	0.6224	0.674	0.447	0.780	0.6619	1.613	0.4297	0.898	0.6075	0.812	0.3465	86	0.972	0.8595
Sweeping																		
every day	148	1.000		1.000		1.000		1.000		1.000		1.000		1.000		144	1.000	
less than every day	156	0.688	0.1404	1.390	0.1481	0.817	0.6404	1.1432	0.779	1.603	0.3553	1.431	**0.0346**	0.918	0.6417	156	0.812	0.1298
Opening hours (per 1 h increase)	304	1.010	0.4752	0.984	0.2000	0.977	0.333	1.011	0.6707	1.028	0.3256	1.004	0.6977	1.008	0.4592	300	1.004	0.5694
Room volume (per 1 m^3^ increase)	304	0.999	0.7050	1.000	0.9338	0.999	0.9198	1.004	0.3852	0.997	0.3901	1.011	**0.0071**	1.000	0.9363	300	1.003	0.2424
Area per employee (per 1 m^2^ increase)	304	0.974	**0.0064**	0.963	**<.0001**	0.9594	**0.0160**	0.968	0.0736	0.952	**0.011**	0.975	**0.0002**	0.991	0.1841	300	0.984	**0.0020**
Conditional ICC^#^		0.36		0.25		0.52		0.63		0.76		0.001		0.26		0.18		

*N*: number of measurements; *β*: regression coefficient; ICC: intraclass correlation coefficient; *P*-values <0.05 are printed in bold.

#: The conditional ICC is a measure of the degree of within-group homogeneity or between-group heterogeneity after controlling for contextual variables.

## Discussion

This is the first study to compare the levels of exposure to allergens from furred animals and selected microbial agents between the workplace and homes of German veterinary practice employees. Although active airborne dust sampling using pumps is the gold standard to assess occupational exposure, we selected EDC for dust sampling for several reasons. Loud noises from the pumps may increase nervousness in animals, thus elevating the risk of injury to staff. Approximately 66% of all reported accidents in veterinary practices are due to scratches, bites, or kicks from animals (Nienhaus *et al.*, 2005). Moreover, using pumps is expensive, needs trained staff for recharging and calibration, and due to short sampling time (2–8 h) increases the probability of obtaining samples with allergen concentrations below the LOD. In addition, passive dust collection with EDC has proven to be a suitable and practical method in other studies (Krop *et al.*, 2014; Schlünssen *et al.*, 2015; Sander *et al.*, 2018), especially if comparative exposure measurements are conducted in homes, which is dependent on study participants who are not trained in exposure measurement techniques.

### Exposure levels in practices and homes

#### Dogs and cats

More than 80% of the animals treated at the veterinary practices were cats and dogs. Accordingly, Can f 1 and Fel d 1 were found in all but one sample from all practices. Samples from employees’ homes with dogs or cats were also positive for Can f 1 and Fel d 1, respectively. The percentage of dog (52%) and cat (38%) ownership in this study was much higher than the average ownership (dogs 19%; cats 23%) in Germany (Zentralverband Zoologischer Fachbetriebe e.V.). In addition, there was also a very high percentage of positive samples in homes without dogs (84%) or cats (66%). In comparison, the positive rate in homes of children and day-care center staff was only 39% (Can f 1—homes without dog) and 27% (Fel d 1—homes without cat) (Sander *et al.*, 2018). Allergen levels were found to be approximately 40-fold (Can f 1) and 100-fold (Fel d 1) higher in homes with pets than in homes without, which agrees with earlier studies examining cat and dog allergens in reservoir dust from floors or mattresses (Custovic *et al.*, 1999; Arbes *et al.*, 2004; Heinrich *et al.*, 2006; Stemeseder *et al.*, 2017). Can f 1 and Fel d 1 values were also elevated in the homes of practice employees with and without cats/dogs compared with the homes of those not occupationally exposed to animals (Krop *et al.*, 2014; Sander *et al.*, 2018). The higher levels in our study may be due to (i) the transfer of allergens from the practice to homes via clothing and hair, (ii) higher number of animals (up to six cats or dogs) at home, and (iii) more frequent and intensive contact between study participants and other pet owners or pets outside their home.

In practices, Can f 1 levels were approximately twice as high as Fel d 1 values (809 versus 440 ng m^−2^), although similar percentages of cats and dogs were examined (mean: 40% for cats and 44% for dogs). One reason is that dogs may release more allergens due to their higher average body mass. Similar Can f 1 (720 ng m^−2^), but lower Fel d 1 (56 ng m^−2^) levels were previously reported in a companion animal hospital using EDCs; however, cats represented only 15% of animals treated in this study (Samadi *et al.*, 2010).

Although concentrations of 1 µg g^−1^ for Fel d 1 and 2 µg g^−1^ for Can f 1 have been associated with allergic sensitization and 8 µg g^−1^ for Fel d 1 and 10 µg g^−1^ with asthma symptoms in sensitized individuals (Salo *et al.*, 2009), these thresholds have not been established so far. Anyway, no such risk levels have been suggested for airborne samples. Nevertheless, it has been demonstrated that respiratory symptoms were induced in sensitized individuals with a brief exposure to airborne levels of Feld 1 that were found in many homes with cats and occasionally in homes without cats (BOLLINGER *et al.*, 1996). For EDC samples, Sander et al. defined exposure levels that predicted the presence of dogs (≥75 ng m^−2^ for Can f 1) and cats (≥46 ng m^−2^ for Fel d 1) in dwellings (Sander *et al.*, 2018). In our study, 280 out of 304 practice rooms had concentrations that exceeded both values, indicating probable health risk for veterinary staff.

#### Rabbits and guinea pigs

This is the first study measuring allergens from rabbits (Ory c 3) and guinea pigs (Cav p 1) using newly developed immunoassays. Both were examined in 35 or 34 of 36 investigated practices and represented 9 and 6% of all treated species, respectively. Accordingly, the percentage of positive samples in practices, and the median allergen levels for both species were lower compared with cats and dogs. The median Cav p 1 level (93 ng m^−2^) was three-times lower than the Ory c 3 level (282 ng m^−2^), which may be related to the smaller surface of guinea pigs compared with rabbits. According to a survey (7000 households) by the Central Association of Zoological Specialized Companies, approx. 5% of households in Germany have a small animal (except cats and dogs) (Zentralverband Zoologischer Fachbetriebe e.V.). Among the practice employees, the percentage of small animal ownership (rabbit, guinea pig, and hamster) was almost twice as high (mean 9%). In all homes with rabbits or guinea pigs, allergens of the respective species could be detected; whereas, the median allergen level was below the LOD in households without. Similar to our study, another allergen Ory c 1 was found in dust from all households with rabbits and was non-detectable in control dwellings (Willerton and Mason, 2018).

#### Horse

The very high percentage of Equ c 1 positive samples in practices and homes (over 70%) was surprising. The possibility that our Equ c 1 assay cross-reacts with the structurally related cat and dog allergens, Fel d 4 and Can f 6 (Hilger *et al.*, 2012; Nilsson *et al.*, 2012; Yamamoto *et al.*, 2019) was excluded by testing recombinant Fel d 4 and Can f 6, as well as cat and dog allergen preparations (data not shown). The specific recognition of Equ c 1 was also supported by showing that employees’ homes with horse contact had considerably higher Equ c 1 values (70-fold) compared with those without. This strongly indicates that allergens can be easily spread to non-animal environments due to passive transfer via clothing or hair. Compared with the allergen levels in homes with pets (1416 ng m^−2^ for Can f 1; 1617 ng m^−2^ for Fel d 1), the relatively high Equ c 1 value (877 ng m^−2^) found in homes of employees in contact with horses indicates that animal size influences amount of allergen transferred.

#### Domestic mite

DM allergen levels were 2–3 times lower in practices than in homes, with or without pets. One reason may be increased cleaning frequency in practices compared with homes, and the absence of upholstered furniture, beds, and carpets—the most important indoor mite habitats (Colloff, 2009; Solarz and Pająk, 2019)—and thus a reservoir for mite allergens (Sander and Neumann et al., 2016). The DM allergen level in practices (median 60 ng m^−2^/2 weeks) was also much lower than the levels measured in Dutch schools (geometric mean 133.5 ng m^−2^/week (Krop *et al.*, 2014)) or German day care centers (median 364 ng m^−2^/2 weeks (Sander *et al.*, 2018)) using the same sampling and quantification methods. No significant difference was seen in DM levels between homes with and without pets (*P* = 0.255), which is consistent with other studies showing that pet presence at homes has no significant effect on DM allergen levels on EDC (Krop *et al.*, 2014; Sander and Neumann *et al.*, 2016; Sander *et al.*, 2018). In contrast, rooms occupied by animals in practices had significantly higher DM allergen levels than those without, probably due to increased use and therefore enhanced air disturbance in these rooms. In undisturbed conditions, airborne mite allergen concentrations are mostly undetectable (Paufler *et al.*, 2001).

#### Endotoxin and β-(1,3)-glucan exposure

In general, direct comparison of endotoxin and β-(1,3)-glucan levels among different studies is often hampered by methodological differences. Measuring both microbial agents can be performed using various quantitative assays (from different manufacturers or laboratories) that produce different nominal values that do not always correlate (Brooks *et al.*, 2013; Liebers *et al.*, 2020). Furthermore, extraction procedure and subsequent storage can influence the results. For example, endotoxin activity in frozen samples was significantly lower than in fresh samples, and adding the detergent Tween 20 to the extraction medium generated significantly higher endotoxin values (Liebers *et al.*, 2007; Spaan *et al.*, 2008). Therefore, comparisons of endotoxin levels are only appropriate within a study. In our study, endotoxin levels were slightly lower in practices than in homes with pets, and twice as high as in homes without pets. Significant difference was obtained between houses with and without pets (*P* = 0.005). This is consistent with other studies reporting that pets (dog or cat) significantly contribute to elevated endotoxin levels in homes (Heinrich *et al.*, 2001; Mendy *et al.*, 2018). This is also seen in the practices, where rooms occupied by animals had significantly higher endotoxin levels than rooms without animals (161 versus 100 EU m^−2^). In the companion animal hospital, highest endotoxin exposure levels were measured in areas with close contact to animals (Samadi *et al.*, 2010).

#### Influences on exposure levels within the practices

Room type significantly influenced exposure levels in practices. Most of the investigated analytes were similarly distributed throughout the practices, with the highest concentrations in examination rooms and the lowest in operating rooms. Examination rooms tend to be used more frequently and for longer periods compared with other rooms with animals. In addition, stress of examination may result in more active animals. Conversely, in operating rooms, animals are usually sedated, and these areas are cleaned and disinfected regularly. Consistent with our results, a Dutch study also found the lowest Can f 1 and Fel d 1 levels in the operating room (Samadi *et al.*, 2010). However, Can f 1 levels were lower in the examination room than the waiting or ultrasound room. In rooms without animals, changing rooms had the highest allergen and endotoxin levels, supporting the hypothesis that clothing is an important allergen carrier. For Equ c 1, the main route of allergen transfer into practices may be via clothing worn by employees who had contact with horses. Pet owners who ride horses may also bring allergens into the practice, explaining the higher levels of Equ c 1 in the waiting room compared with the examination room. Significantly high levels of cow hair allergens were also discovered in cattle farmers’ changing rooms due to allergen transfer from stables (Zahradnik *et al.*, 2011). Apart from room type, exposure was strongly influenced by the area per employee. Allergen and β-(1,3)-glucan levels decreased with lower occupancy, which may be due to lower air and dust turbulence. This is supported by higher allergen levels measured in break rooms (used by all employees) compared with individual offices (mainly used by only one person). More frequent ventilation (per window) did not reduce exposure levels. In fact, slightly lower exposure was observed in rooms that were never ventilated, although the difference was significant only for Fel d 1, endotoxin, and β-(1,3)-glucan. This may be also explained by lower dust turbulence in non-ventilated rooms. Finally, more frequent cleaning measures had no influence on exposure levels, but the differences between ‘cleaning every day’ and ‘less than every day’—which we used for grouping—might be rather small.

## Conclusions

Our data demonstrated that high exposures to animal allergens occur in certain areas of veterinary practices, while the mite allergen levels were low. Although the animal allergen concentrations in veterinary practices were lower than in households with the corresponding animals, it cannot be excluded that such levels are sufficient to cause symptoms in allergic patients. A practical option to reduce the allergen levels could be the use of air filtration systems or portable air cleaners with HEPA filters. To prevent the transfer of allergens from the workplace to the home and vice versa, it would be important to avoid contamination of clothing as much as possible.

## Supplementary Material

wxab053_suppl_Supplementary_file

## Data Availability

The data underlying this article are available in the article and in its online supplementary material.
