# Supplementary material for: Animal Allergens, Endotoxin, and β-(1,3)-Glucan in Small Animal Practices: Exposure Levels at Work and in Homes of Veterinary Staff
Source: Ann Work Expo Health. 2021 Aug 7;66(1):27–40. doi: 10.1093/annweh/wxab053 (PMC8751790; doi:10.1093/annweh/wxab053)
Supplement: wxab053_suppl_Supplementary_file [file wxab053_suppl_supplementary_file.docx]

**Animal allergens, endotoxin and β-(1,3)-glucan in small animal practices: Exposure levels at work and in homes of veterinary staff**

Eva Zahradnik^1^*, Ingrid Sander^1^, Olaf Kleinmüller^2^, Anne Lotz^1^, Verena Liebers^1^, Bente Janssen-Weets^3,4^, Stéphanie Kler^3^, Christiane Hilger^3^, Alexandra Beine^1^, Frank Hoffmeyer^1^, Albert Nienhaus^2,5^, Monika Raulf^1^

^1^Institute for Prevention and Occupational Medicine of the German Social Accident Insurance, Institute of the Ruhr-Universität Bochum (IPA), Bochum, Germany

^2^CVcare, Universitätsklinikum Hamburg-Eppendorf, Hamburg, Germany

^3^Department of Infection and Immunity, Luxemburg Institute of Health, Esch-sur-Alzette, Luxemburg

^4^Department of Dermatology and Allergy Center, Odense Research Center for Anaphylaxis, University of Southern Denmark, Odense, Denmark

^5^ Department of Occupational Medicine, Hazardous Substances and Health Research (AGG), Institution for Statutory Accident Insurance and Prevention in the Health and Welfare Services (BGW), Hamburg, Germany

* Author to whom correspondence should be addressed. E-mail: zahradnik@ipa-dguv.de

**Expression and purification of recombinant Ory c 3 and Cav p 1**

Native Ory c 3 is a dimer consisting of 2 chains, Ory c 3.A.0101 and Ory c 3.B.0101. The recombinant protein was expressed from a construct containing both open reading frames in a head-to-tail configuration. Ory c 3.A.0101 (accession no Q9GK63) was fused to Ory c 3.B.0101 (accession no Q9GK67), followed by a 6 histidine tag. Codons were optimized for expression in *E. coli*. Recombinant Ory c 3 was expressed in Rosetta Gami 2 (DE3) (Merck, Darmstadt, Germany) and purified by immobilized metal ion affinity chromatography (HisTrap HP, GE Healthcare) under native conditions according to the manufacturer’s instructions. Eluted fractions containing the recombinant protein were pooled and dialyzed and further purifed by anion exchange chromatography (Resource Q column, GE Healthcare) using a 0 to 500 mM NaCl gradient in 20 mM Bis-Tris HCl (pH 7).

The cDNA sequences coding for the mature proteins Cav p 1.0101 and Cav p 1.0201 (GenBank accession no VEV85353 and VEV85354) were subcloned into pQE-60 (Qiagen) and expressed in *E.coli* Rosetta Gami 2 (DE3) as recombinant proteins with a C-terminal Hexa-Histidine tag. The recombinant proteins were purified under native conditions as described above. The second purification step was done by ion exchange chromatography using a RESOURCE Q column a linear gradient of 0-500 mM NaCl in 20mM TRIS-HCl, pH8.

**Production, purification and biotinylation of polyclonal antibodies**

## Polyclonal antibodies (pAbs) to Ory c 3 and Cav p 1 were generated by a commercial provider (Eurogentec; Seraing, Belgium) and total IgG was obtained. For Ory c 3, guinea pigs were immunized by 4 antigen injections of 60 µg recombinant 6xHis-tagged Ory c 3, according to the company’s standardized protocol. Anti-Cav p 1 polyclonal antibodies were obtained using a similar protocol by immunization of rabbits with 200 µg Cav p 1 per injection, consisting of a 1:1 mixture of recombinant and His-tagged isoallergens Cav p 1.0101 and Cav p 1.0201. A part of each purified total IgG batch was labeled with biotin by incubation with 33-fold molar excess of EZ-link NHS-LC-Biotin (Thermo Scientific, Rockford, IL, USA) according to the manufacturer's instructions. The labeled antibodies were then dialyzed against PBS.
